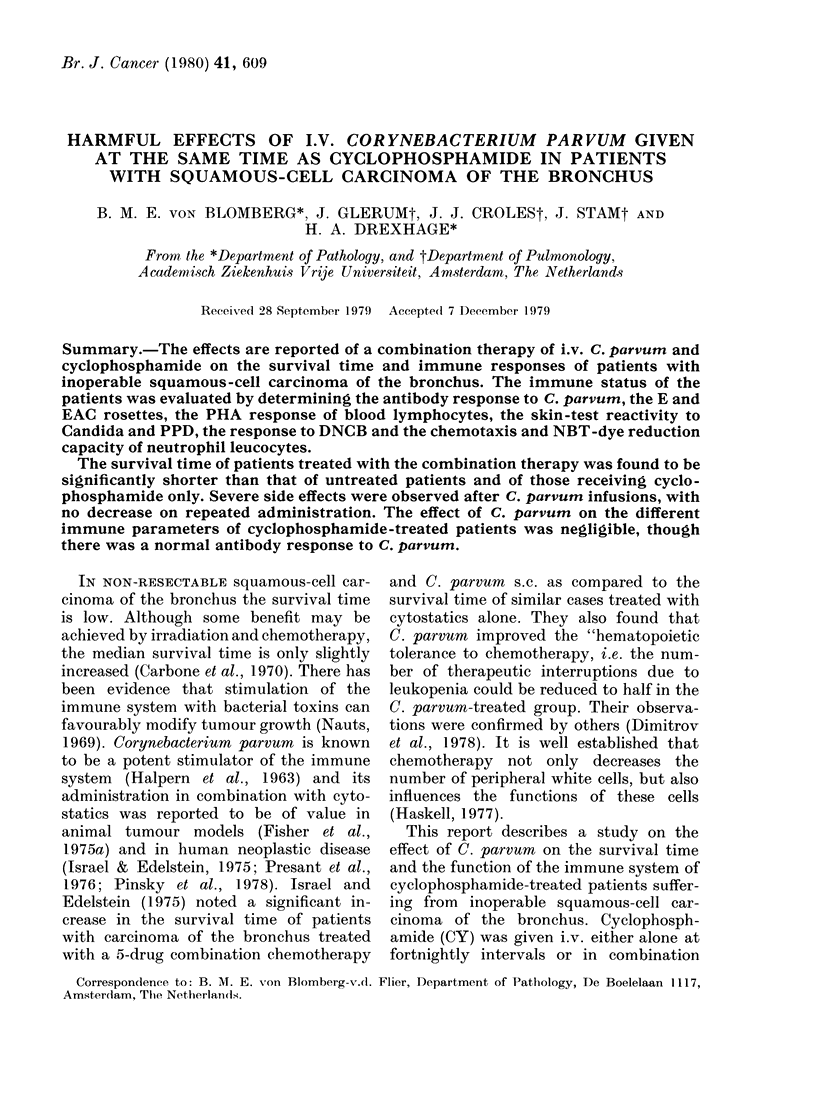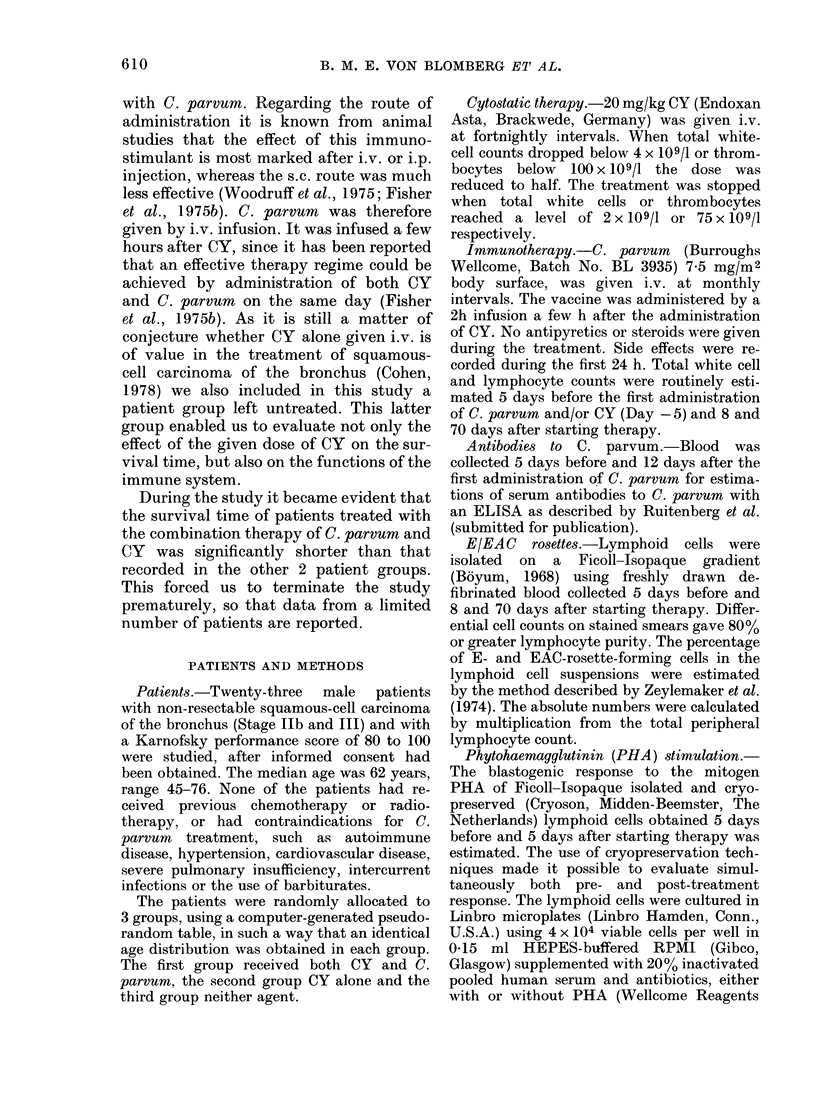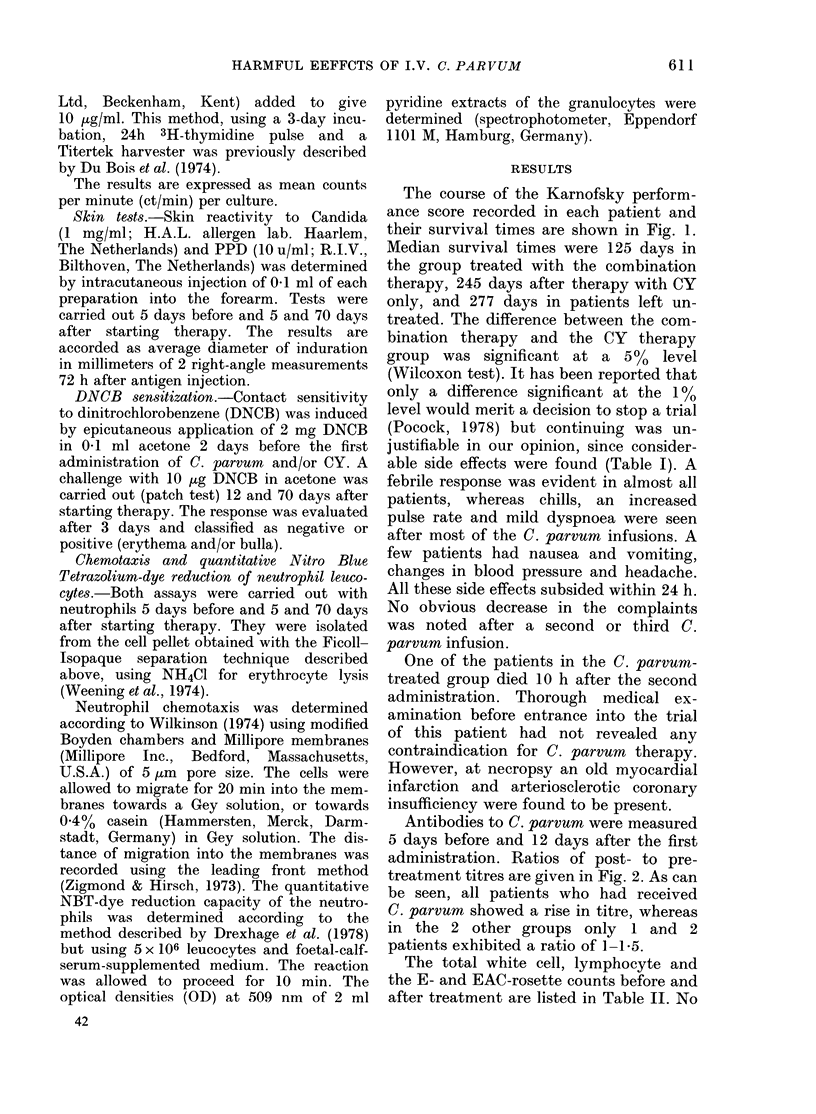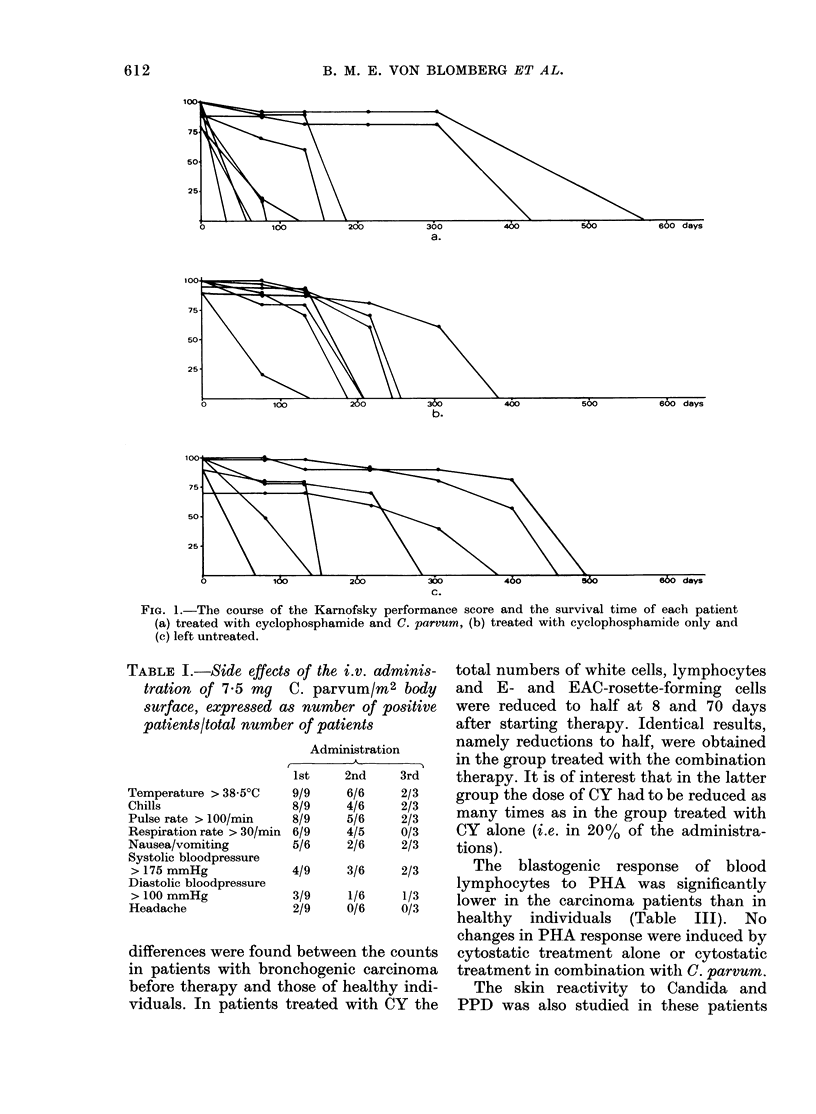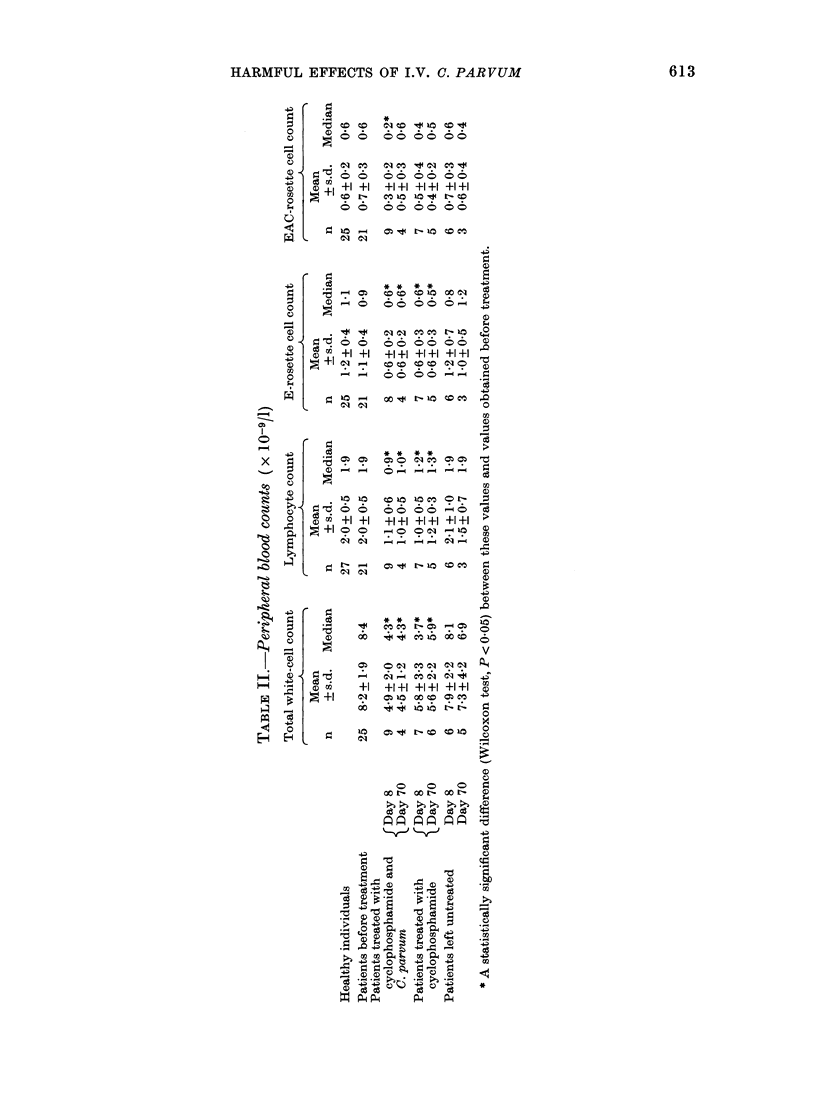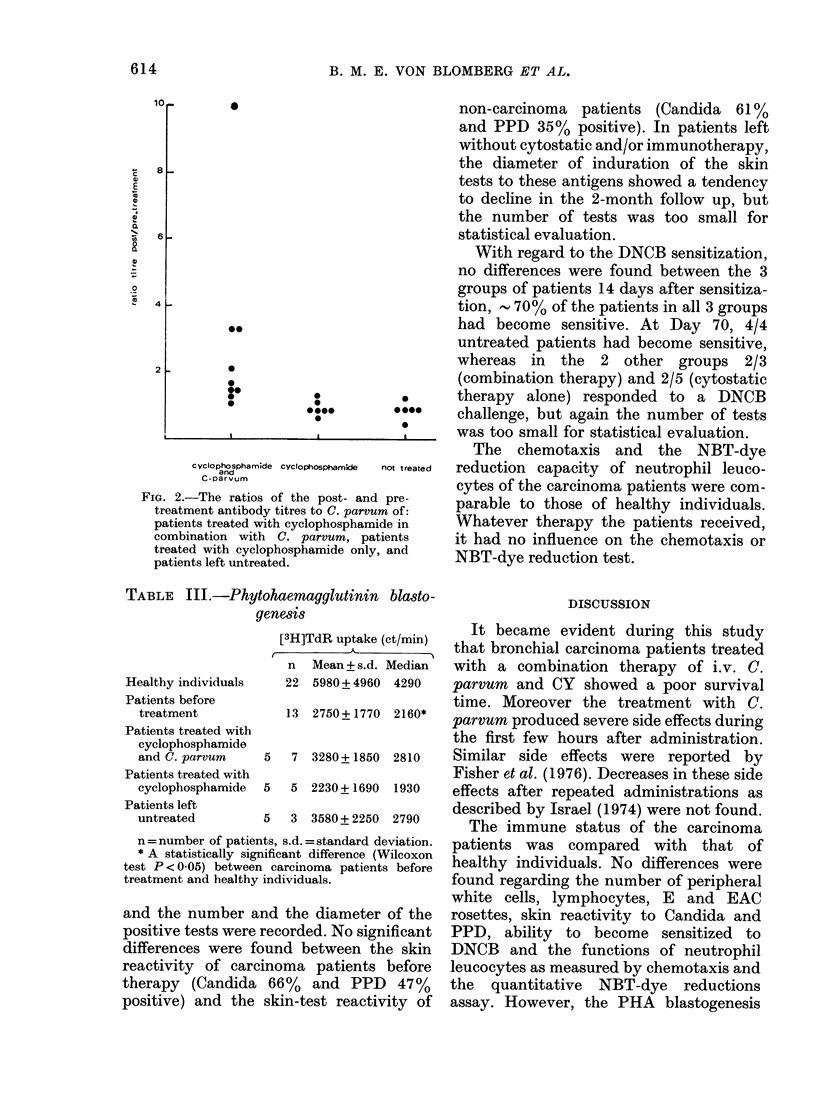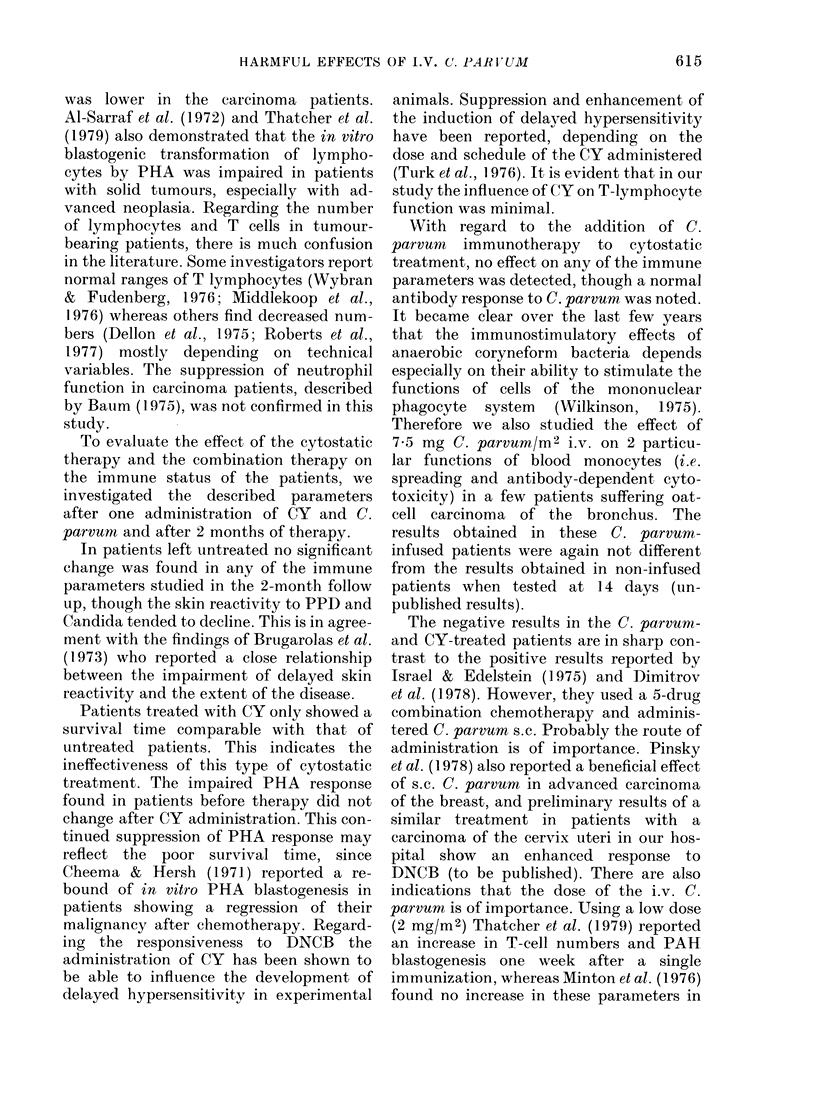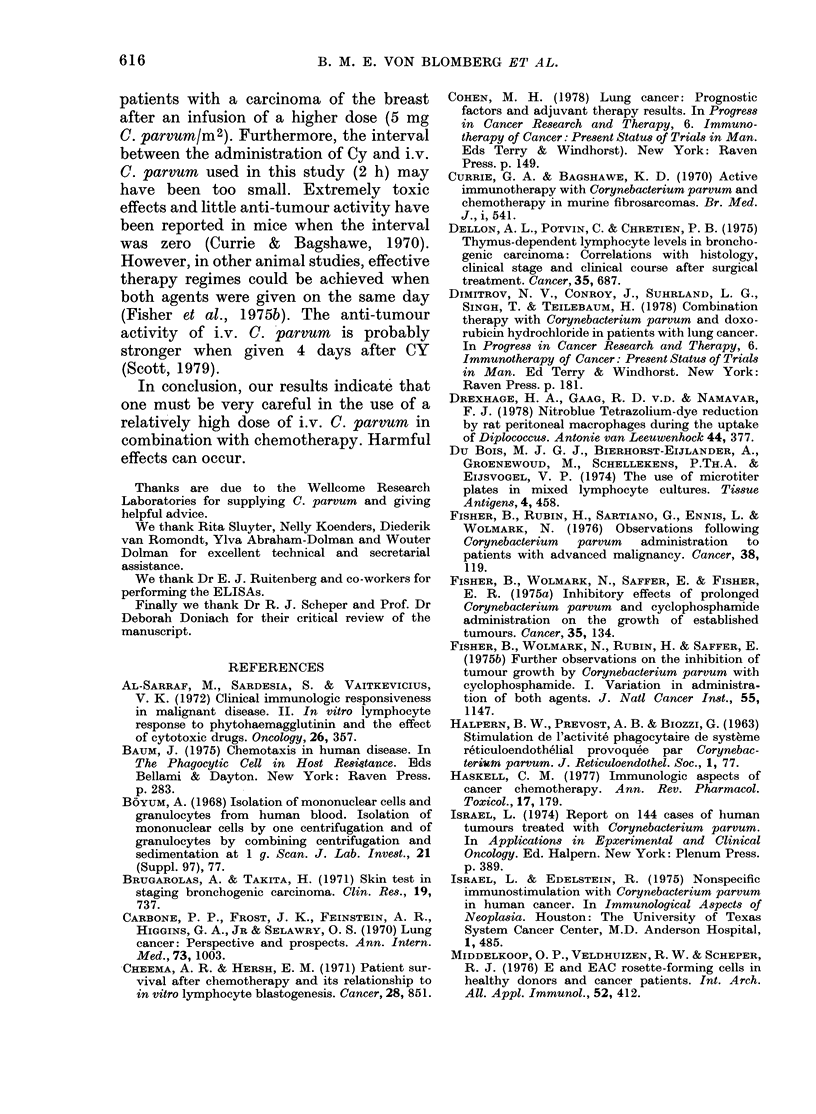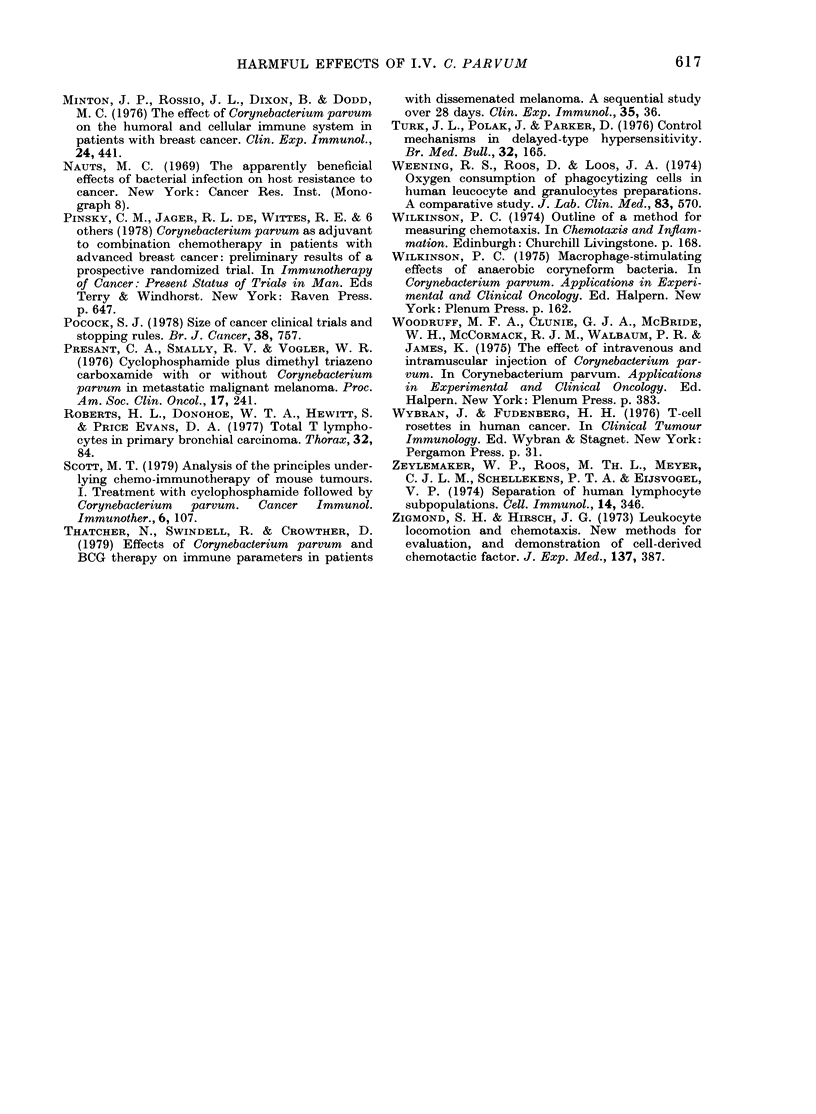# Harmful effects of i.v. Corynebacterium Parvum given at the same time as cyclophosphamide in patients with squamous-cell carcinoma of the bronchus.

**DOI:** 10.1038/bjc.1980.104

**Published:** 1980-04

**Authors:** B. M. von Blomberg, J. Glerum, J. J. Croles, J. Stam, H. A. Drexhage

## Abstract

The effects are reported of a combination therapy of i.v. C. parvum and cyclophosphamide on the survival time and immune responses of patients with inoperable squamous-cell carcinoma of the bronchus. The immune status of the patients was evaluated by determining the antibody response to C. parvum, the E and EAC rosettes, the PHA response of blood lymphocytes, the skin-test reactivity to Candida and PPD, the response to DNCB and the chemotaxis and NBT-dye reduction capacity of neutrophil leucocytes. The survival time of patients treated with the combination therapy was found to be significantly shorter than that of untreated patients and of those receiving cyclophosphamide only. Severe side effects were observed after C. parvum infusions, with no decrease on repeated administration. The effect of C. parvum on the different immune parameters of cyclophosphamide-treated patients was negligible, though there was a normal antibody response to C. parvum.


Br. J. Cancer (1980) 41, 609

### HARMFUL EFFECTS OF I.V. *CORYNEBACTERIUM PARVUM* GIVEN AT THE SAME TIME AS CYCLOPHOSPHAMIDE IN PATIENTS WITH SQUAMOUS-CELL CARCINOMA OF THE BRONCHUS

B. M. E. von BLOMBERG*, J. GLERUM†, J. J. CROLES†, J. STAM† and H. A. DREXHAGE*

*From the *Department of Pathology, and †Department of Pulmonology, Academisch Ziekenhuis Vrije Universiteit, Amsterdam, The Netherlands*

Received 28 September 1979 Accepted 7 December 1979

**Summary.—The effects are reported of a combination therapy of i.v. *C. parvum* and cyclophosphamide on the survival time and immune responses of patients with inoperable squamous-cell carcinoma of the bronchus. The immune status of the patients was evaluated by determining the antibody response to *C. parvum*, the E and EAC rosettes, the PHA response of blood lymphocytes, the skin-test reactivity to Candida and PPD, the response to DNCB and the chemotaxis and NBT-dye reduction capacity of neutrophil leucocytes.**

**The survival time of patients treated with the combination therapy was found to be significantly shorter than that of untreated patients and of those receiving cyclophosphamide only. Severe side effects were observed after *C. parvum* infusions, with no decrease on repeated administration. The effect of *C. parvum* on the different immune parameters of cyclophosphamide-treated patients was negligible, though there was a normal antibody response to *C. parvum*.**

In NON-RESECTABLE squamous-cell carcinoma of the bronchus the survival time is low. Although some benefit may be achieved by irradiation and chemotherapy, the median survival time is only slightly increased (Carbone *et al.*, 1970). There has been evidence that stimulation of the immune system with bacterial toxins can favourably modify tumour growth (Nauts, 1969). *Corynebacterium parvum* is known to be a potent stimulator of the immune system (Halpern *et al.*, 1963) and its administration in combination with cytostatics was reported to be of value in animal tumour models (Fisher *et al.*, 1975*a*) and in human neoplastic disease (Israel & Edelstein, 1975; Presant *et al.*, 1976; Pinsky *et al.*, 1978). Israel and Edelstein (1975) noted a significant increase in the survival time of patients with carcinoma of the bronchus treated with a 5-drug combination chemotherapy and *C. parvum* s.c. as compared to the survival time of similar cases treated with cytostatics alone. They also found that *C. parvum* improved the "hematopoietic tolerance to chemotherapy, *i.e.* the number of therapeutic interruptions due to leukopenia could be reduced to half in the *C. parvum*-treated group. Their observations were confirmed by others (Dimitrov *et al.*, 1978). It is well established that chemotherapy not only decreases the number of peripheral white cells, but also influences the functions of these cells (Haskell, 1977).

This report describes a study on the effect of *C. parvum* on the survival time and the function of the immune system of cyclophosphamide-treated patients suffering from inoperable squamous-cell carcinoma of the bronchus. Cyclophosphamide (CY) was given i.v. either alone at fortnightly intervals or in combination

Correspondence to: B. M. E. von Blomberg-v.d. Flier, Department of Pathology, De Boelelaan 1117, Amsterdam, The Netherlands.

with *C. parvum*. Regarding the route of administration it is known from animal studies that the effect of this immunostimulant is most marked after i.v. or i.p. injection, whereas the s.c. route was much less effective (Woodruff *et al.*, 1975; Fisher *et al.*, 1975*b*). *C. parvum* was therefore given by i.v. infusion. It was infused a few hours after CY, since it has been reported that an effective therapy regime could be achieved by administration of both CY and *C. parvum* on the same day (Fisher *et al.*, 1975*b*). As it is still a matter of conjecture whether CY alone given i.v. is of value in the treatment of squamous-cell carcinoma of the bronchus (Cohen, 1978) we also included in this study a patient group left untreated. This latter group enabled us to evaluate not only the effect of the given dose of CY on the survival time, but also on the functions of the immune system.

During the study it became evident that the survival time of patients treated with the combination therapy of *C. parvum* and CY was significantly shorter than that recorded in the other 2 patient groups. This forced us to terminate the study prematurely, so that data from a limited number of patients are reported.

### PATIENTS AND METHODS

*Patients.*—Twenty-three male patients with non-resectable squamous-cell carcinoma of the bronchus (Stage IIb and III) and with a Karnofsky performance score of 80 to 100 were studied, after informed consent had been obtained. The median age was 62 years, range 45–76. None of the patients had received previous chemotherapy or radiotherapy, or had contraindications for *C. parvum* treatment, such as autoimmune disease, hypertension, cardiovascular disease, severe pulmonary insufficiency, intercurrent infections or the use of barbiturates.

The patients were randomly allocated to 3 groups, using a computer-generated pseudorandom table, in such a way that an identical age distribution was obtained in each group. The first group received both CY and *C. parvum*, the second group CY alone and the third group neither agent.

*Cytostatic therapy.*—20 mg/kg CY (Endoxan Asta, Brackwede, Germany) was given i.v. at fortnightly intervals. When total white-cell counts dropped below $4 \times 10^9$/l or thrombocytes below $100 \times 10^9$/l the dose was reduced to half. The treatment was stopped when total white cells or thrombocytes reached a level of $2 \times 10^9$/l or $75 \times 10^9$/l respectively.

*Immunotherapy.*—*C. parvum* (Burroughs Wellcome, Batch No. BL 3935) 7·5 mg/m$^2$ body surface, was given i.v. at monthly intervals. The vaccine was administered by a 2h infusion a few h after the administration of CY. No antipyretics or steroids were given during the treatment. Side effects were recorded during the first 24 h. Total white cell and lymphocyte counts were routinely estimated 5 days before the first administration of *C. parvum* and/or CY (Day −5) and 8 and 70 days after starting therapy.

*Antibodies to* C. parvum.—Blood was collected 5 days before and 12 days after the first administration of *C. parvum* for estimations of serum antibodies to *C. parvum* with an ELISA as described by Ruitenberg *et al.* (submitted for publication).

*E/EAC rosettes.*—Lymphoid cells were isolated on a Ficoll–Isopaque gradient (Böyum, 1968) using freshly drawn defibrinated blood collected 5 days before and 8 and 70 days after starting therapy. Differential cell counts on stained smears gave 80% or greater lymphocyte purity. The percentage of E- and EAC-rosette-forming cells in the lymphoid cell suspensions were estimated by the method described by Zeylemaker *et al.* (1974). The absolute numbers were calculated by multiplication from the total peripheral lymphocyte count.

*Phytohaemagglutinin (PHA) stimulation.*—The blastogenic response to the mitogen PHA of Ficoll–Isopaque isolated and cryopreserved (Cryoson, Midden-Beemster, The Netherlands) lymphoid cells obtained 5 days before and 5 days after starting therapy was estimated. The use of cryopreservation techniques made it possible to evaluate simultaneously both pre- and post-treatment response. The lymphoid cells were cultured in Linbro microplates (Linbro Hamden, Conn., U.S.A.) using $4 \times 10^4$ viable cells per well in 0·15 ml HEPES-buffered RPMI (Gibco, Glasgow) supplemented with 20% inactivated pooled human serum and antibiotics, either with or without PHA (Wellcome Reagents

Ltd, Beckenham, Kent) added to give 10 μg/ml. This method, using a 3-day incubation, 24h $^{3}$H-thymidine pulse and a Titertek harvester was previously described by Du Bois *et al.* (1974).

The results are expressed as mean counts per minute (ct/min) per culture.

*Skin tests.*—Skin reactivity to Candida (1 mg/ml; H.A.L. allergen lab. Haarlem, The Netherlands) and PPD (10 u/ml; R.I.V., Bilthoven, The Netherlands) was determined by intracutaneous injection of 0·1 ml of each preparation into the forearm. Tests were carried out 5 days before and 5 and 70 days after starting therapy. The results are accorded as average diameter of induration in millimeters of 2 right-angle measurements 72 h after antigen injection.

*DNCB sensitization.*—Contact sensitivity to dinitrochlorobenzene (DNCB) was induced by epicutaneous application of 2 mg DNCB in 0·1 ml acetone 2 days before the first administration of *C. parvum* and/or CY. A challenge with 10 μg DNCB in acetone was carried out (patch test) 12 and 70 days after starting therapy. The response was evaluated after 3 days and classified as negative or positive (erythema and/or bulla).

*Chemotaxis and quantitative Nitro Blue Tetrazolium-dye reduction of neutrophil leucocytes.*—Both assays were carried out with neutrophils 5 days before and 5 and 70 days after starting therapy. They were isolated from the cell pellet obtained with the Ficoll–Isopaque separation technique described above, using $NH_4Cl$ for erythrocyte lysis (Weening *et al.*, 1974).

Neutrophil chemotaxis was determined according to Wilkinson (1974) using modified Boyden chambers and Millipore membranes (Millipore Inc., Bedford, Massachusetts, U.S.A.) of 5 μm pore size. The cells were allowed to migrate for 20 min into the membranes towards a Gey solution, or towards 0·4% casein (Hammersten, Merck, Darmstadt, Germany) in Gey solution. The distance of migration into the membranes was recorded using the leading front method (Zigmond & Hirsch, 1973). The quantitative NBT-dye reduction capacity of the neutrophils was determined according to the method described by Drexhage *et al.* (1978) but using $5 \times 10^{6}$ leucocytes and foetal-calf-serum-supplemented medium. The reaction was allowed to proceed for 10 min. The optical densities (OD) at 509 nm of 2 ml pyridine extracts of the granulocytes were determined (spectrophotometer, Eppendorf 1101 M, Hamburg, Germany).

### RESULTS

The course of the Karnofsky performance score recorded in each patient and their survival times are shown in Fig. 1. Median survival times were 125 days in the group treated with the combination therapy, 245 days after therapy with CY only, and 277 days in patients left untreated. The difference between the combination therapy and the CY therapy group was significant at a 5% level (Wilcoxon test). It has been reported that only a difference significant at the 1% level would merit a decision to stop a trial (Pocock, 1978) but continuing was unjustifiable in our opinion, since considerable side effects were found (Table I). A febrile response was evident in almost all patients, whereas chills, an increased pulse rate and mild dyspnoea were seen after most of the *C. parvum* infusions. A few patients had nausea and vomiting, changes in blood pressure and headache. All these side effects subsided within 24 h. No obvious decrease in the complaints was noted after a second or third *C. parvum* infusion.

One of the patients in the *C. parvum*-treated group died 10 h after the second administration. Thorough medical examination before entrance into the trial of this patient had not revealed any contraindication for *C. parvum* therapy. However, at necropsy an old myocardial infarction and arteriosclerotic coronary insufficiency were found to be present.

Antibodies to *C. parvum* were measured 5 days before and 12 days after the first administration. Ratios of post- to pre-treatment titres are given in Fig. 2. As can be seen, all patients who had received *C. parvum* showed a rise in titre, whereas in the 2 other groups only 1 and 2 patients exhibited a ratio of 1–1·5.

The total white cell, lymphocyte and the E- and EAC-rosette counts before and after treatment are listed in Table II. No

FIG. 1.—The course of the Karnofsky performance score and the survival time of each patient (a) treated with cyclophosphamide and *C. parvum*, (b) treated with cyclophosphamide only and (c) left untreated.

TABLE I.—*Side effects of the i.v. administration of* 7·5 *mg* C. parvum/*m*[2] *body surface, expressed as number of positive patients/total number of patients*

| | Administration | | |
|---|---|---|---|
| | 1st | 2nd | 3rd |
| Temperature > 38·5°C | 9/9 | 6/6 | 2/3 |
| Chills | 8/9 | 4/6 | 2/3 |
| Pulse rate > 100/min | 8/9 | 5/6 | 2/3 |
| Respiration rate > 30/min | 6/9 | 4/5 | 0/3 |
| Nausea/vomiting | 5/6 | 2/6 | 2/3 |
| Systolic bloodpressure > 175 mmHg | 4/9 | 3/6 | 2/3 |
| Diastolic bloodpressure > 100 mmHg | 3/9 | 1/6 | 1/3 |
| Headache | 2/9 | 0/6 | 0/3 |

differences were found between the counts in patients with bronchogenic carcinoma before therapy and those of healthy individuals. In patients treated with CY the total numbers of white cells, lymphocytes and E- and EAC-rosette-forming cells were reduced to half at 8 and 70 days after starting therapy. Identical results, namely reductions to half, were obtained in the group treated with the combination therapy. It is of interest that in the latter group the dose of CY had to be reduced as many times as in the group treated with CY alone (*i.e.* in 20% of the administrations).

The blastogenic response of blood lymphocytes to PHA was significantly lower in the carcinoma patients than in healthy individuals (Table III). No changes in PHA response were induced by cytostatic treatment alone or cytostatic treatment in combination with *C. parvum*.

The skin reactivity to Candida and PPD was also studied in these patients

TABLE II.—*Peripheral blood counts* ( $\times 10^{-9}$/l)

| | | Total white-cell count | | | Lymphocyte count | | | E-rosette cell count | | | EAC-rosette cell count | | |
|---|---|---|---|---|---|---|---|---|---|---|---|---|---|
| | | n | Mean ±s.d. | Median | n | Mean ±s.d. | Median | n | Mean ±s.d. | Median | n | Mean ±s.d. | Median |
| Healthy individuals | | | | | 27 | 2·0±0·5 | 1·9 | 25 | 1·2±0·4 | 1·1 | 25 | 0·6±0·2 | 0·6 |
| Patients before treatment | | 25 | 8·2±1·9 | 8·4 | 21 | 2·0±0·5 | 1·9 | 21 | 1·1±0·4 | 0·9 | 21 | 0·7±0·3 | 0·6 |
| Patients treated with cyclophosphamide and *C. parvum* | Day 8 | 9 | 4·9±2·0 | 4·3* | 9 | 1·1±0·6 | 0·9* | 8 | 0·6±0·2 | 0·6* | 9 | 0·3±0·2 | 0·2* |
| | Day 70 | 4 | 4·5±1·2 | 4·3* | 4 | 1·0±0·5 | 1·0* | 4 | 0·6±0·2 | 0·6* | 4 | 0·5±0·3 | 0·6 |
| Patients treated with cyclophosphamide | Day 8 | 7 | 5·8±3·3 | 3·7* | 7 | 1·0±0·5 | 1·2* | 7 | 0·6±0·3 | 0·6* | 7 | 0·5±0·4 | 0·4 |
| | Day 70 | 6 | 5·6±2·2 | 5·9* | 5 | 1·2±0·3 | 1·3* | 5 | 0·6±0·3 | 0·5* | 5 | 0·4±0·2 | 0·5 |
| Patients left untreated | Day 8 | 6 | 7·9±2·2 | 8·1 | 6 | 2·1±1·0 | 1·9 | 6 | 1·2±0·7 | 0·8 | 6 | 0·7±0·3 | 0·6 |
| | Day 70 | 5 | 7·3±4·2 | 6·9 | 3 | 1·5±0·7 | 1·9 | 3 | 1·0±0·5 | 1·2 | 3 | 0·6±0·4 | 0·4 |

* A statistically significant difference (Wilcoxon test, $P < 0{\cdot}05$) between these values and values obtained before treatment.

FIG. 2.—The ratios of the post- and pre-treatment antibody titres to *C. parvum* of: patients treated with cyclophosphamide in combination with *C. parvum*, patients treated with cyclophosphamide only, and patients left untreated.

TABLE III.—*Phytohaemagglutinin blastogenesis*

| | | [$^3$H]TdR uptake (ct/min) | | |
|---|---|---|---|---|
| | | n | Mean ± s.d. | Median |
| Healthy individuals | | 22 | 5980 ± 4960 | 4290 |
| Patients before treatment | | 13 | 2750 ± 1770 | 2160* |
| Patients treated with cyclophosphamide and *C. parvum* | 5 | 7 | 3280 ± 1850 | 2810 |
| Patients treated with cyclophosphamide | 5 | 5 | 2230 ± 1690 | 1930 |
| Patients left untreated | 5 | 3 | 3580 ± 2250 | 2790 |

n = number of patients, s.d. = standard deviation.
* A statistically significant difference (Wilcoxon test $P < 0{\cdot}05$) between carcinoma patients before treatment and healthy individuals.

and the number and the diameter of the positive tests were recorded. No significant differences were found between the skin reactivity of carcinoma patients before therapy (Candida 66% and PPD 47% positive) and the skin-test reactivity of non-carcinoma patients (Candida 61% and PPD 35% positive). In patients left without cytostatic and/or immunotherapy, the diameter of induration of the skin tests to these antigens showed a tendency to decline in the 2-month follow up, but the number of tests was too small for statistical evaluation.

With regard to the DNCB sensitization, no differences were found between the 3 groups of patients 14 days after sensitization, ~70% of the patients in all 3 groups had become sensitive. At Day 70, 4/4 untreated patients had become sensitive, whereas in the 2 other groups 2/3 (combination therapy) and 2/5 (cytostatic therapy alone) responded to a DNCB challenge, but again the number of tests was too small for statistical evaluation.

The chemotaxis and the NBT-dye reduction capacity of neutrophil leucocytes of the carcinoma patients were comparable to those of healthy individuals. Whatever therapy the patients received, it had no influence on the chemotaxis or NBT-dye reduction test.

### DISCUSSION

It became evident during this study that bronchial carcinoma patients treated with a combination therapy of i.v. *C. parvum* and CY showed a poor survival time. Moreover the treatment with *C. parvum* produced severe side effects during the first few hours after administration. Similar side effects were reported by Fisher *et al.* (1976). Decreases in these side effects after repeated administrations as described by Israel (1974) were not found.

The immune status of the carcinoma patients was compared with that of healthy individuals. No differences were found regarding the number of peripheral white cells, lymphocytes, E and EAC rosettes, skin reactivity to Candida and PPD, ability to become sensitized to DNCB and the functions of neutrophil leucocytes as measured by chemotaxis and the quantitative NBT-dye reductions assay. However, the PHA blastogenesis was lower in the carcinoma patients. Al-Sarraf *et al.* (1972) and Thatcher *et al.* (1979) also demonstrated that the *in vitro* blastogenic transformation of lymphocytes by PHA was impaired in patients with solid tumours, especially with advanced neoplasia. Regarding the number of lymphocytes and T cells in tumour-bearing patients, there is much confusion in the literature. Some investigators report normal ranges of T lymphocytes (Wybran & Fudenberg, 1976; Middlekoop *et al.*, 1976) whereas others find decreased numbers (Dellon *et al.*, 1975; Roberts *et al.*, 1977) mostly depending on technical variables. The suppression of neutrophil function in carcinoma patients, described by Baum (1975), was not confirmed in this study.

To evaluate the effect of the cytostatic therapy and the combination therapy on the immune status of the patients, we investigated the described parameters after one administration of CY and *C. parvum* and after 2 months of therapy.

In patients left untreated no significant change was found in any of the immune parameters studied in the 2-month follow up, though the skin reactivity to PPD and Candida tended to decline. This is in agreement with the findings of Brugarolas *et al.* (1973) who reported a close relationship between the impairment of delayed skin reactivity and the extent of the disease.

Patients treated with CY only showed a survival time comparable with that of untreated patients. This indicates the ineffectiveness of this type of cytostatic treatment. The impaired PHA response found in patients before therapy did not change after CY administration. This continued suppression of PHA response may reflect the poor survival time, since Cheema & Hersh (1971) reported a rebound of *in vitro* PHA blastogenesis in patients showing a regression of their malignancy after chemotherapy. Regarding the responsiveness to DNCB the administration of CY has been shown to be able to influence the development of delayed hypersensitivity in experimental animals. Suppression and enhancement of the induction of delayed hypersensitivity have been reported, depending on the dose and schedule of the CY administered (Turk *et al.*, 1976). It is evident that in our study the influence of CY on T-lymphocyte function was minimal.

With regard to the addition of *C. parvum* immunotherapy to cytostatic treatment, no effect on any of the immune parameters was detected, though a normal antibody response to *C. parvum* was noted. It became clear over the last few years that the immunostimulatory effects of anaerobic coryneform bacteria depends especially on their ability to stimulate the functions of cells of the mononuclear phagocyte system (Wilkinson, 1975). Therefore we also studied the effect of 7·5 mg *C. parvum*/m$^2$ i.v. on 2 particular functions of blood monocytes (*i.e.* spreading and antibody-dependent cytotoxicity) in a few patients suffering oat-cell carcinoma of the bronchus. The results obtained in these *C. parvum*-infused patients were again not different from the results obtained in non-infused patients when tested at 14 days (unpublished results).

The negative results in the *C. parvum*- and CY-treated patients are in sharp contrast to the positive results reported by Israel & Edelstein (1975) and Dimitrov *et al.* (1978). However, they used a 5-drug combination chemotherapy and administered *C. parvum* s.c. Probably the route of administration is of importance. Pinsky *et al.* (1978) also reported a beneficial effect of s.c. *C. parvum* in advanced carcinoma of the breast, and preliminary results of a similar treatment in patients with a carcinoma of the cervix uteri in our hospital show an enhanced response to DNCB (to be published). There are also indications that the dose of the i.v. *C. parvum* is of importance. Using a low dose (2 mg/m$^2$) Thatcher *et al.* (1979) reported an increase in T-cell numbers and PAH blastogenesis one week after a single immunization, whereas Minton *et al.* (1976) found no increase in these parameters in patients with a carcinoma of the breast after an infusion of a higher dose (5 mg *C. parvum*/m$^2$). Furthermore, the interval between the administration of Cy and i.v. *C. parvum* used in this study (2 h) may have been too small. Extremely toxic effects and little anti-tumour activity have been reported in mice when the interval was zero (Currie & Bagshawe, 1970). However, in other animal studies, effective therapy regimes could be achieved when both agents were given on the same day (Fisher *et al.*, 1975*b*). The anti-tumour activity of i.v. *C. parvum* is probably stronger when given 4 days after CY (Scott, 1979).

In conclusion, our results indicate that one must be very careful in the use of a relatively high dose of i.v. *C. parvum* in combination with chemotherapy. Harmful effects can occur.

Thanks are due to the Wellcome Research Laboratories for supplying *C. parvum* and giving helpful advice.

We thank Rita Sluyter, Nelly Koenders, Diederik van Romondt, Ylva Abraham-Dolman and Wouter Dolman for excellent technical and secretarial assistance.

We thank Dr E. J. Ruitenberg and co-workers for performing the ELISAs.

Finally we thank Dr R. J. Scheper and Prof. Dr Deborah Doniach for their critical review of the manuscript.

### REFERENCES

AL-SARRAF, M., SARDESIA, S. & VAITKEVICIUS, V. K. (1972) Clinical immunologic responsiveness in malignant disease. II. *In vitro* lymphocyte response to phytohaemagglutinin and the effect of cytotoxic drugs. *Oncology*, **26**, 357.

BAUM, J. (1975) Chemotaxis in human disease. In *The Phagocytic Cell in Host Resistance*. Eds Bellami & Dayton. New York: Raven Press. p. 283.

BÖYUM, A. (1968) Isolation of mononuclear cells and granulocytes from human blood. Isolation of mononuclear cells by one centrifugation and of granulocytes by combining centrifugation and sedimentation at 1 *g*. *Scan. J. Lab. Invest.*, **21** (Suppl. 97), 77.

BRUGAROLAS, A. & TAKITA, H. (1971) Skin test in staging bronchogenic carcinoma. *Clin. Res.*, **19**, 737.

CARBONE, P. P., FROST, J. K., FEINSTEIN, A. R., HIGGINS, G. A., JR & SELAWRY, O. S. (1970) Lung cancer: Perspective and prospects. *Ann. Intern. Med.*, **73**, 1003.

CHEEMA, A. R. & HERSH, E. M. (1971) Patient survival after chemotherapy and its relationship to *in vitro* lymphocyte blastogenesis. *Cancer*, **28**, 851.

COHEN, M. H. (1978) Lung cancer: Prognostic factors and adjuvant therapy results. In *Progress in Cancer Research and Therapy*, 6. *Immunotherapy of Cancer: Present Status of Trials in Man.* Eds Terry & Windhorst). New York: Raven Press. p. 149.

CURRIE, G. A. & BAGSHAWE, K. D. (1970) Active immunotherapy with *Corynebacterium parvum* and chemotherapy in murine fibrosarcomas. *Br. Med. J.*, i, 541.

DELLON, A. L., POTVIN, C. & CHRETIEN, P. B. (1975) Thymus-dependent lymphocyte levels in bronchogenic carcinoma: Correlations with histology, clinical stage and clinical course after surgical treatment. *Cancer*, **35**, 687.

DIMITROV, N. V., CONROY, J., SUHRLAND, L. G., SINGH, T. & TEILEBAUM, H. (1978) Combination therapy with *Corynebacterium parvum* and doxorubicin hydrochloride in patients with lung cancer. In *Progress in Cancer Research and Therapy*, 6. *Immunotherapy of Cancer: Present Status of Trials in Man.* Ed Terry & Windhorst. New York: Raven Press. p. 181.

DREXHAGE, H. A., GAAG, R. D. V.D. & NAMAVAR, F. J. (1978) Nitroblue Tetrazolium-dye reduction by rat peritoneal macrophages during the uptake of *Diplococcus. Antonie van Leeuwenhock* **44**, 377.

DU BOIS, M. J. G. J., BIERHORST-EIJLANDER, A., GROENEWOUD, M., SCHELLEKENS, P.TH.A. & EIJSVOGEL, V. P. (1974) The use of microtiter plates in mixed lymphocyte cultures. *Tissue Antigens*, **4**, 458.

FISHER, B., RUBIN, H., SARTIANO, G., ENNIS, L. & WOLMARK, N. (1976) Observations following *Corynebacterium parvum* administration to patients with advanced malignancy. *Cancer*, **38**, 119.

FISHER, B., WOLMARK, N., SAFFER, E. & FISHER, E. R. (1975*a*) Inhibitory effects of prolonged *Corynebacterium parvum* and cyclophosphamide administration on the growth of established tumours. *Cancer*, **35**, 134.

FISHER, B., WOLMARK, N., RUBIN, H. & SAFFER, E. (1975*b*) Further observations on the inhibition of tumour growth by *Corynebacterium parvum* with cyclophosphamide. I. Variation in administration of both agents. *J. Natl Cancer Inst.*, **55**, 1147.

HALPERN, B. W., PREVOST, A. B. & BIOZZI, G. (1963) Stimulation de l'activité phagocytaire de système réticuloendothélial provoquée par *Corynebacterium parvum. J. Reticuloendothel. Soc.*, **1**, 77.

HASKELL, C. M. (1977) Immunologic aspects of cancer chemotherapy. *Ann. Rev. Pharmacol. Toxicol.*, **17**, 179.

ISRAEL, L. (1974) Report on 144 cases of human tumours treated with *Corynebacterium parvum*. In *Applications in Epxerimental and Clinical Oncology*. Ed. Halpern. New York: Plenum Press. p. 389.

ISRAEL, L. & EDELSTEIN, R. (1975) Nonspecific immunostimulation with *Corynebacterium parvum* in human cancer. In *Immunological Aspects of Neoplasia*. Houston: The University of Texas System Cancer Center, M.D. Anderson Hospital, **1**, 485.

MIDDELKOOP, O. P., VELDHUIZEN, R. W. & SCHEPER, R. J. (1976) E and EAC rosette-forming cells in healthy donors and cancer patients. *Int. Arch. All. Appl. Immunol.*, **52**, 412.

MINTON, J. P., ROSSIO, J. L., DIXON, B. & DODD, M. C. (1976) The effect of *Corynebacterium parvum* on the humoral and cellular immune system in patients with breast cancer. *Clin. Exp. Immunol.*, **24,** 441.

NAUTS, M. C. (1969) The apparently beneficial effects of bacterial infection on host resistance to cancer. New York: Cancer Res. Inst. (Monograph 8).

PINSKY, C. M., JAGER, R. L. DE, WITTES, R. E. & 6 others (1978) *Corynebacterium parvum* as adjuvant to combination chemotherapy in patients with advanced breast cancer: preliminary results of a prospective randomized trial. In *Immunotherapy of Cancer: Present Status of Trials in Man.* Eds Terry & Windhorst. New York: Raven Press. p. 647.

POCOCK, S. J. (1978) Size of cancer clinical trials and stopping rules. *Br. J. Cancer*, **38,** 757.

PRESANT, C. A., SMALLY, R. V. & VOGLER, W. R. (1976) Cyclophosphamide plus dimethyl triazeno carboxamide with or without *Corynebacterium parvum* in metastatic malignant melanoma. *Proc. Am. Soc. Clin. Oncol.*, **17,** 241.

ROBERTS, H. L., DONOHOE, W. T. A., HEWITT, S. & PRICE EVANS, D. A. (1977) Total T lymphocytes in primary bronchial carcinoma. *Thorax*, **32,** 84.

SCOTT, M. T. (1979) Analysis of the principles underlying chemo-immunotherapy of mouse tumours. I. Treatment with cyclophosphamide followed by *Corynebacterium parvum. Cancer Immunol. Immunother.*, **6,** 107.

THATCHER, N., SWINDELL, R. & CROWTHER, D. (1979) Effects of *Corynebacterium parvum* and BCG therapy on immune parameters in patients with dissemenated melanoma. A sequential study over 28 days. *Clin. Exp. Immunol.*, **35,** 36.

TURK, J. L., POLAK, J. & PARKER, D. (1976) Control mechanisms in delayed-type hypersensitivity. *Br. Med. Bull.*, **32,** 165.

WEENING, R. S., ROOS, D. & LOOS, J. A. (1974) Oxygen consumption of phagocytizing cells in human leucocyte and granulocytes preparations. A comparative study. *J. Lab. Clin. Med.*, **83,** 570.

WILKINSON, P. C. (1974) Outline of a method for measuring chemotaxis. In *Chemotaxis and Inflammation.* Edinburgh: Churchill Livingstone. p. 168.

WILKINSON, P. C. (1975) Macrophage-stimulating effects of anaerobic coryneform bacteria. In *Corynebacterium parvum. Applications in Experimental and Clinical Oncology.* Ed. Halpern. New York: Plenum Press. p. 162.

WOODRUFF, M. F. A., CLUNIE, G. J. A., MCBRIDE, W. H., MCCORMACK, R. J. M., WALBAUM, P. R. & JAMES, K. (1975) The effect of intravenous and intramuscular injection of *Corynebacterium parvum.* In Corynebacterium parvum. *Applications in Experimental and Clinical Oncology.* Ed. Halpern. New York: Plenum Press. p. 383.

WYBRAN, J. & FUDENBERG, H. H. (1976) T-cell rosettes in human cancer. In *Clinical Tumour Immunology.* Ed. Wybran & Stagnet. New York: Pergamon Press. p. 31.

ZEYLEMAKER, W. P., ROOS, M. TH. L., MEYER, C. J. L. M., SCHELLEKENS, P. T. A. & EIJSVOGEL, V. P. (1974) Separation of human lymphocyte subpopulations. *Cell. Immunol.*, **14,** 346.

ZIGMOND, S. H. & HIRSCH, J. G. (1973) Leukocyte locomotion and chemotaxis. New methods for evaluation, and demonstration of cell-derived chemotactic factor. *J. Exp. Med.*, **137,** 387.